# Topological phase structures of conical refraction beams: expanding orbital angular momentum applications for nanoscale biosensing

**DOI:** 10.1515/nanoph-2025-0511

**Published:** 2025-11-25

**Authors:** Diana Galiakhmetova, Nawal Mohamed, Fatima Khanom, Shakti Singh, Gennadii Piavchenko, Grigorii S. Sokolovskii, Edik Rafailov, Igor Meglinski

**Affiliations:** Aston Institute of Photonic Technologies, 1722 Aston University, Birmingham, UK; Human Anatomy and Histology Department, I. M. Sechenov First Moscow State Medical University (Sechenov University), Moscow, Russia; A.F. Ioffe Physico-Technical Institute, Russian Academy of Sciences, Saint Petersburg, Russia

**Keywords:** conical refraction, orbital angular momentum, turbid scattering medium, cancer diagnostics

## Abstract

Topologically structured light carrying orbital angular momentum (OAM) has emerged as a powerful tool for nano-photonics and biomedical optics, yet conventional integer-charge Laguerre–Gaussian (LG) beams suffer from rotational degeneracy that limits diagnostic precision. Here, we demonstrate that conical refraction (CR) beams, specifically the Lloyd, Poggendorff, and Raman families, overcome this fundamental limitation through their inherent generation of fractional OAM states with unambiguous phase signatures. Through systematic interferometric comparison of LG (*ℓ* = 3, 5) and CR beam propagation in tissues, we show that CR beams achieve superior diagnostic performance: while LG beams exhibit three-fold rotational ambiguity (4.19 rad uncertainty), Poggendorff CR beams provide phase determination with 0.08 rad precision. Both LG and CR beam families display remarkable topological resilience, preserving phase coherence as they traverse tissue samples while attaining refractive index sensitivity at the 10^−6^ level, three orders of magnitude beyond conventional refractometry. Most significantly, we present the first experimental evidence that CR beams can discriminate between healthy and cancerous kidney tissues through distinct phase rotations (4.71 vs. 5.04 rad, *p* < 0.001) and a tenfold amplification in polarisation-induced distortion. The fractional topological charges of CR beams, ranging continuously between integer values, expand the accessible OAM phase space and enable 3.7-fold superior signal-to-noise ratio compared to ${\text{LG}}_{0}^{3}$ measurements. These results establish CR-generated fractional OAM as the preferred modality for label-free tissue diagnostics, bridging fundamental nanophotonics with clinical applications in cancer detection and intraoperative margin assessment.

## 1 Introduction

The rise of *topologically structured light* has significantly transformed modern photonics, establishing orbital angular momentum (OAM) as a cornerstone for both nanophotonics and biomedical optics [1], [2], [3]. Since the seminal identification of helical phase beams as carriers of OAM by Allen et al. [4], Laguerre–Gaussian (LG) beams have served as the canonical model of topological light [5]. Yet, the field has rapidly advanced far beyond LG modes, embracing a rich family of structured beams, including Bessel, Hermite–Laguerre–Gaussian, Mathieu, Airy beams [6], and conical refraction (CR) beams [7], [8], that unlock new topological phase landscapes with unique physical properties.

Recent breakthroughs have demonstrated that OAM beams preserve their phase even when propagating through highly scattering tissues [9], [10], directly contradicting the long-held belief that optical coherence is fragile in complex media [11]. This robustness, rooted in the topological nature of OAM, enables refractive index sensitivity at the 10^−6^ level and opens new opportunities for quantum- and nano-photonics enhanced biomedical optical diagnostics [11], [12]. From super-resolution imaging and label-free cancer detection to the conceptual development of a ‘quantum biopsy’, OAM light is no longer an optical curiosity but a disruptive tool defining the frontier of quantum biophotonics and translational diagnostics.

Within this landscape, CR beams occupy a special position: they provide compact, broadband, and power-resilient sources of fractional OAM states, offering precise topological control and unambiguous phase singularities [7]. In the current study, we explore the topological phase structures of CR beams and benchmark their performance against LG modes in scattering media, paving the way toward nano-photonic-based scales for high-sensitivity biomedical sensing and early disease detection.

### 1.1 Application of structured light with OAM

**Figure 1:**
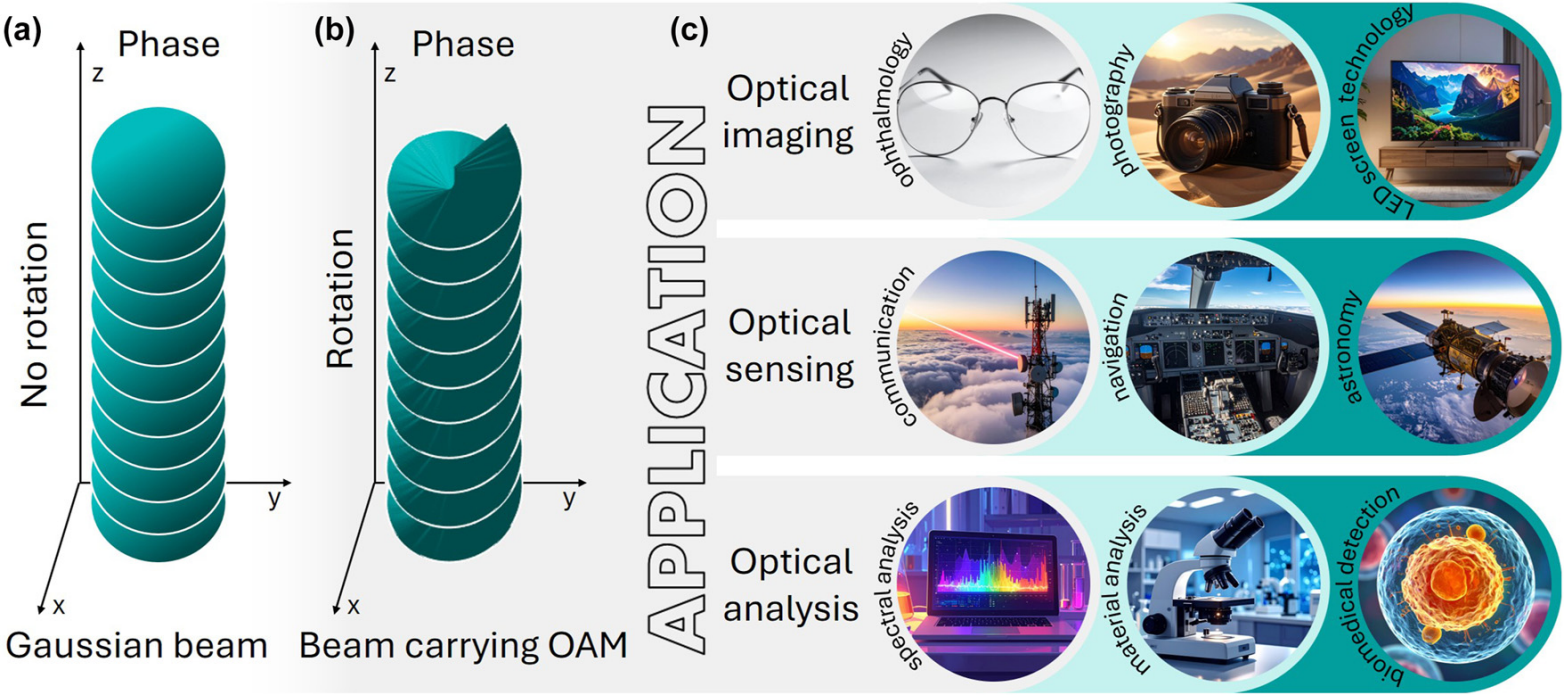
Schematic illustration of the spatial phase evolution for a Gaussian beam (a), a beam carrying OAM (b), and representative application areas of structured light carrying OAM (c).

Structured light is attracting intense interest because its non-classical properties, such as phase topologies, OAM, and spatially varying polarisation, enable functionalities inaccessible to conventional beams [13], [14]. Unlike Gaussian beams (Figure 1a), light carrying OAM has a helical wavefront that rotates during propagation while preserving its spiral phase (Figure 1b). This unique property finds applications in a wide range of fields (see Figure 1c), offering the possibility of using it as a novel alternative for current tools based on classical light property to advance optical communication [15], [16], [17], imaging and sensing [18], [19], particles and cells tweezing [20], [21], and biomedical analysis and detection [9], [22], [23].

Beams carrying OAM, such as LG [9], [24], Bessel [25], Bessel–Gaussian [8], [26], Hermite–Gaussian [27], [28], hypergeometric [29], and perfect vortex [30], are typically generated using spiral phase plates (SPPs) [31], [32], spatial light modulators (SLMs) [9], hologram [16], or non-linear metasurfaces [33]. Despite their widespread and long-standing use, they exhibit certain limitations: a fixed topological charge for a specific wavelength, low damage thresholds (<0.1 J/cm^2^) or bulk cooling systems and complex fabrication processes [17], [34].

### 1.2 Conical refraction as OAM carrier

**Figure 2:**
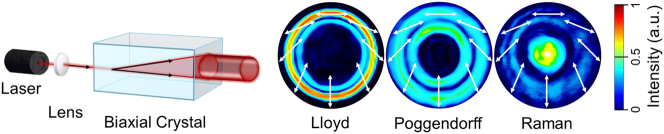
Schematic representation of internal CR: a collimated light beam passing through a biaxial crystal parallel to one of its optical axes refracts inside the crystal and emerges as a hollow light cylinder. The resulting cylinder exhibits distinct intensity beam profiles (Lloyd, Poggendorff, and Raman) and polarisation distributions, which vary with the position of the observation camera.

In contrast to a uniaxial crystal, which possesses two principal refractive indices and exhibits birefringence, splitting light into two orthogonally polarized components (ordinary and extraordinary beams), a biaxial crystal (BC) has three distinct principal refractive indices and two optical axes. When light propagates through a BC, it also undergoes birefringence; however, instead of splitting into two separate beams, the light spreads conically within the crystal and emerges as a hollow cylindrical beam behind the exit facet of the BC [35]. This phenomenon is called CR. Since the light propagates as a dual cone inside the biaxial crystal [7], it produces a characteristic intensity distribution that may appear as a double ring of nearly equal size (Lloyd), two rings of comparable intensity (Poggendorff), or a single ring with a central bright spot (Raman) (Figure 2). These distinct beam patterns were named after their discoverers: Humphrey Lloyd (1832), Johann Poggendorff (1839), and Sir C. V. Raman (1940), respectively. The formation of these three patterns can be controlled by adjusting the propagation distance of the focused light within the crystal.

An especially compelling feature of CR is its natural ability to generate beams with fractional OAM [36]. Unlike canonical LG modes that are restricted to integer topological charges, CR beams inherently support non-integer charge states with precisely defined phase singularities. These fractional OAM states expand the accessible topological phase space, offering unique advantages for both fundamental nano-photonics and biomedical optics, where they can act as highly sensitive probes of refractive index variations and subtle tissue abnormalities.

Despite nearly two centuries of research into CR [37], the OAM content and phase evolution of its characteristic beam families, i.e. Lloyd, Poggendorff, and Raman, have remained only partially explored and are still not fully understood. To our knowledge, this is the first systematic of their topological phase structures, demonstrating that all three CR patterns carry OAM and retain their robustness when propagating through both normal and cancerous tissue samples. Cancer progression is rooted in nano-scale morphological and biochemical alterations, which manifest optically as subtle refractive index and polarization changes. The demonstrated sensitivity of CR family and LG-based OAM beams to such variations situates this approach squarely within the domain of nanophotonics, highlighting its dual relevance for both fundamental photonics research and translational biomedical applications. By benchmarking their performance against canonical LG beams, we establish CR-based beams as a previously unexplored class of topologically structured light with unique advantages for refractive index sensing and disease detection. These results set the stage for developing nano-photonic-scale diagnostics that leverage fractional OAM states as sensitive, label-free probes of biological complexity.

## 2 Materials and methods

### 2.1 Experimental setups

**Figure 3:**
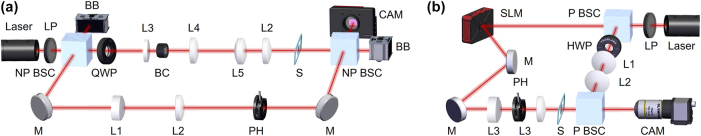
Schematic presentation of the Mach–Zehnder interferometer experimental setups for CR family beams (a): NP BSC non-polarising beamsplitter cube, LP linear polarizer, BB beam block, QWP quarter-wave plate, BC biaxial crystal, L1, L2, L3, L4, L5 lenses, M mirror, PH pinhole, S sample, CAM camera; and for LG beams (b): P BSC polarising beamsplitter cube, HWP half-wave plate, L1, L2, L3 lenses.

The CR family and LG-based beams carrying OAM were generated using 6 mm-long Nd (3 at.%) doped KGd(WO_4_)_2_ biaxial crystal (BC) and SLM – spatial light modulator (PLUTO-2-NIR-011, Holoeye, Germany), respectively. Mach-Zehnder interferometers (Figure 3) with continuous-wave 633 nm HeNe lasers (Thorlabs, HRR020-1, USA and BioRay laser diode, Coherent, USA) were used to characterize the phase of the structured beams. The laser beams passed through a linear polarizer LP (Thorlabs, USA) and were split into two arms using non-polarized (Eksma Optics, Lithuania) and polarizing (Thorlabs, USA) beam splitter cubes for the CR and LG Mach-Zehnder setups, respectively.

In the case of CR-structured beams (see Figure 3a), a Gaussian beam (1 mm-diameter) passing through a quarter-wave plate QWP (SAQWP05M-1700, Thorlabs, USA), was separated into reference and sample arms. The reference arm (bottom) included mirrors M, plano-convex lenses L1 (*f* = 20 mm), L2 (*f* = 120 mm) to expand the Gaussian beam, and a pinhole PH to cut the beam to diameter 10 mm. The sample arm (up) included the plano-convex lens L3 (*f* = 25 mm) and BC. The Gaussian beam was converted into a Lloyd, Poggendorff, or Raman ring depending on the position of plano-convex lens L4 (*f* = 150 mm). After the system of optical lenses L5 (*f* = 150 mm) and L2, the 3 mm-diameter CR-structured beam was focused on the sample S and then collinearly interfered with a plane wave Gaussian beam from the reference arm and recorded on camera CAM (CS135MU, Thorlabs, USA).

In the case of the LG-structured beam (see Figure 3b), the Gaussian beam was split by a polarising beam splitter cube P BSC (Thorlabs, USA). The reference arm (right) had a half-wave plate HWP, and two lenses L1 (*f* = 30 mm) and L2 (*f* = 75 mm). The sample arm included the SLM, system of mirrors M, pinhole PH, and two lenses L3 (*f* = 45 mm) (Thorlabs, USA) to direct and focus the LG beam with a corresponding forked diffraction pattern to sample (beam diameter 3 mm). The LG beam was interfered with plane wave of 10 mm-diameter expanded Gaussian beam passing through the second P BSC and recorded on camera CAM (DCC3240M, Thorlabs, USA).

In both experimental studies, the phase retrieval was carried out using a fast Fourier transform (FFT)-based method [9], with normalization to the diameter of the beam to ensure fair comparisons. The initial intensity level and beam size for the different samples were adjusted using a gradual neutral density filter and monitored by the camera.

### 2.2 Sample preparation

For the validation of the refractive index sensitivity of the LG beam, an ethanol-water solution with a water concentration of 51.89 mol was prepared. The solution was filled into the 1 mm-thick quartz cuvette (100-1-20, Hellma Analytics, Germany). Before the experiments, the sample was cooled down to 13 °C in a fridge. During the measurements, the room temperature was set on a constant value of 23 °C, while the temperature of sample was controlled and constantly recorded by thermocouple (PicoLog TC-08, Pico Technology, UK) and thermal camera (C5, Teledyne FLIR, USA). The temperature measurement resolution of the thermocouple is 0.025 °C. The temperature and concentration dependence of refractive index values of ethanol-water solution were used from the literature [38].

For the study of cancerous and healthy tissues, we used a 5 μm-thick transparent medium, histological specimens (*n* = 6 per sample) from samples of kidney (*n* = 6 per both healthy and malignant groups). The specimens were placed between standard microscope slides and cover slips. All measurements were repeated three times at different locations (fields of view) on each sample, with 10 consecutive recordings per location, using a setting with one camera profile recording every second.

### 2.3 Phase retrieval technique

**Figure 4:**
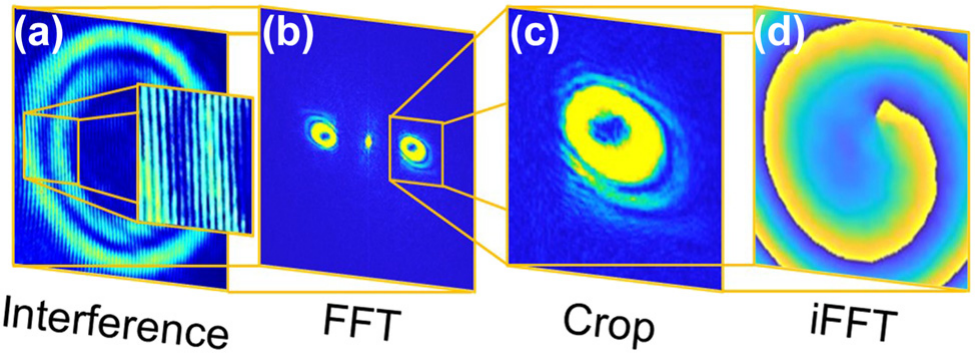
Schematic presentation of phase retrieval process for CR- and LG-based structured beams [9]. Recorded off-axis interference pattern (a), FFT of the captured interference (b), crop of the frequency spectrum corresponding to the structured beam (c), and iFFT of the cropped spectrum (d).

The phase distribution of the different structured beams based on the CR and LG families was obtained using a straightforward signal processing approach [9], [39]. In the first step (Figure 4a), the off-axis Mach–Zehnder interference pattern of the sample and reference arms (see Figure 3) was recorded using a CCD camera. Next, the recorded interference pattern was processed using a FFT to extract the corresponding frequency spectrum (see Figure 4b). A careful selection with maximum pixel resolution was then applied to isolate the relevant frequency components corresponding to the CR or LG beam and to minimise background noise arising from backscattered light interference (see Figure 4c). Finally, the cropped FFT image was subjected to an inverse fast Fourier transform (iFFT), resulting in the phase distribution of the specific structured beam (see Figure 4d).

## 3 Results and discussion

### 3.1 Structured light OAM phase sensitivity

**Figure 5:**
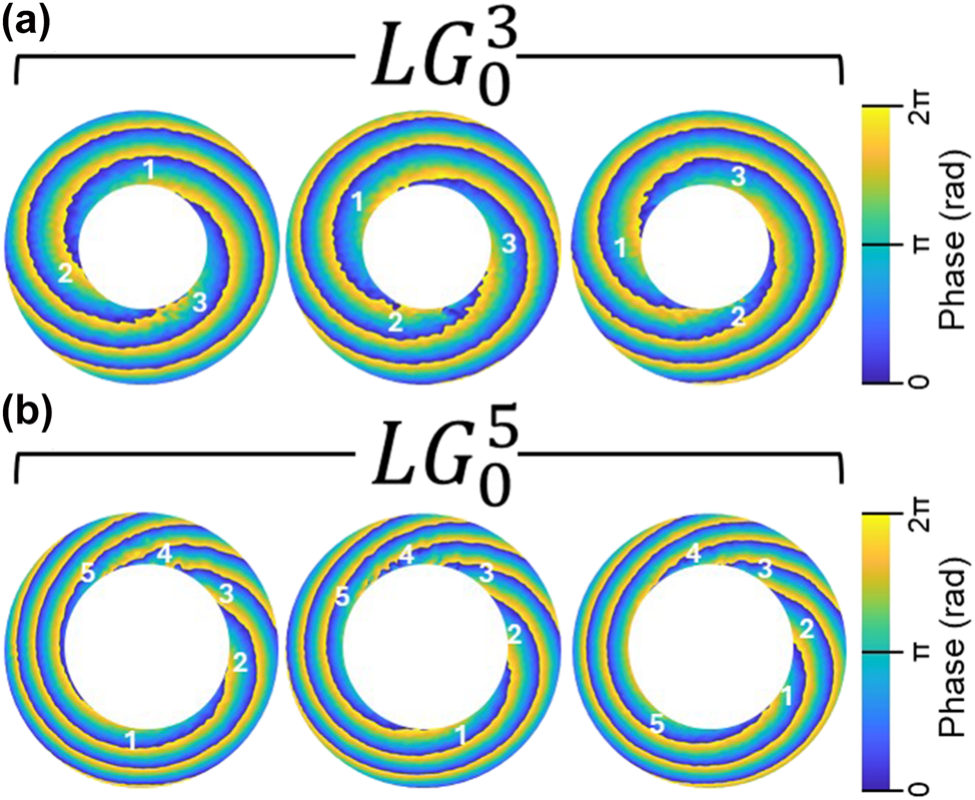
Phase twists of OAM of the LG beams with topological charges *l* = 3 (a) and *l* = 5 (b) during the gradual increase of refractive index (5 × 10^−6^). The sample is 1 mm-thick cuvette containing an ethanol solution with a water concentration of 51.89 mol%.

The unique ability of OAM to preserve its spiral phase structure while propagating through tissue-like medium [9], [10] opens the possibility of using it as a tool to detect minute changes in refractive index in tissue samples studies [12]. Conventional diagnostic methods, limited to refractive index sensitivities of ∼10^−3^, are rapidly being outpaced by OAM-based approaches, where non-classical light properties such as OAM enable resolution down to 10^−6^. In our experiments within current study, such sensitivity is demonstrated using LG beams with topological charges of *ℓ* = 3 and *ℓ* = 5 (Figure 5). We used a 1 mm-thick cuvette filled with an ethanol–water solution with a water concentration of 51.89 mol%. During a gradual increase in the solution temperature from 13 °C up to 23 °C (room temperature), we continuously measured the sample temperature and recorded the corresponding beam intensity profile using a CCD camera. Then, we recovered the phase using a FFT-based method and identified the minimal visible changes in the vortex positions for ${\text{LG}}_{0}^{3}$ and ${\text{LG}}_{0}^{5}$ . Figure 5 shows the rotation of each vortex as a function of temperature (22.25 °C, 22.38 °C, and 22.52 °C) and the corresponding refractive index values (1.363916 [left], 1.363868 [middle], 1.363816 [right]) of the sampling medium. The observed vortex rotation corresponds to a three-order-of-magnitude improvement in refractive index sensitivity compared to conventional methods. Such an extraordinary sensitivity of OAM light to refractive index variations arises from the intrinsic coupling between spiral trajectories within its helical phase structure. As OAM beams propagate within a medium, photons follow spiral-like trajectories of different effective path lengths across the beam profile. A tiny change in refractive index Δ*n* modifies the accumulated scalar phase of the OAM arm:

(1) $$\Delta \Psi \approx k_{0}\;\Delta n\;L_{\text{eff}},$$

where *k*_0_ = 2*π*/*λ*_0_ and *L*_eff_ is the helical energy–flow path of the OAM beam set by its azimuthal Poynting component. In interferometric readouts (OAM vs. plane-wave/Gaussian reference), a global phase shift of the OAM arm appears as a rigid rotation of the 2|*ℓ*|-petal pattern by

(2) Δθ=ΔΨ/ℓ,

thus transducing minute Δ*n* into a readily measurable angular rotation [9].

Therefore, even minute changes in the medium’s refractive index alter the effective optical path length of OAM beams, producing a cumulative azimuthal phase shift that appears as a coherent twist of the helical wavefront [9]. When spatial gradients of *n*(**r**) are present, they further couple to the azimuthally varying phase structure, imprinting an additional phase shear that enhances the distinguishability of refractive index variations in complex samples. Unlike classical light fields, where scattering rapidly randomizes phase, the topological phase memory of OAM preserves these twists even in a turbid tissue, enabling detection of perturbations as small as 10^−6^. This unique combination of geometric amplification and topological protection establishes OAM beams as exceptionally sensitive probes of subtle biochemical and structural changes in biological media.

### 3.2 Topological phase landscapes of CR beam families

**Figure 6:**
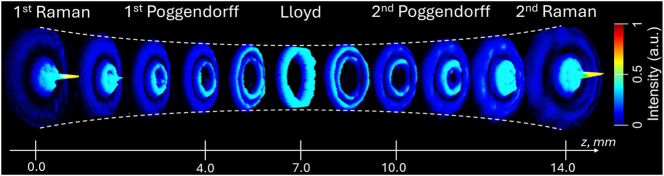
Spatial evolution of the CR-structured light intensity recorded at the sample plane as a function of the position of the plano-convex lens *L*4 (*f* = 150 mm), with the initial lens position set to *z* = 0 at the 1st Raman ring.

When a Gaussian beam propagates through a BC, it transforms into two conical waves, manifesting as a series of Raman and Poggendorff rings on both sides of the central Lloyd ring. Our experimental results on switching between different CR–structured beams, including the so-called 1st Raman, 1st Poggendorff, Lloyd, 2nd Poggendorff, and 2nd Raman, demonstrated that these profiles can be easily adjusted by varying the position of a plano-convex lens *L*4 (*f* = 150 mm) along the *z*-axis. Moving the lens by 4.0 mm, 7.0 mm, 10.0 mm, and 14 mm (with the 1st Raman position taken as the reference at *z* = 0) allows precise control over the output pattern. The spectral evolution of the CR-structured light intensity and profile as a function of the plano-convex lens position is shown in Figure 6.

#### 3.2.1 Diffractive theory of CR beams family

The unique optical beam profilic patterns utilised in our experiments, such as the Raman spot, the Poggendorff dark ring, and the Lloyd-based cylindrical ring structure (Figure 6), are all direct creations of the CR phenomenon. To quantify and model the formation alongside the propagation of these beams, the simplistic geometric ray optics strategy was extended to describe and employ the full diffractive theory of CR, as elegantly reformulated by Berry [40] from the works of Belsky and Khapalyuk [41]. This formalism is necessary for predicting the intricate intensity profile and phase singularities that demonstrate their unique propogation, particularly through biological media.

The diffractive theory treats a BC as a unitary transformation operator $\hat{U}$ acting on the angular spectrum of the input beam [7], [42]. For an input electric field **E**_in_(**r**), its 2D Fourier transform yields the plane-wave spectrum $A\left(k\right)={\left[A_{x}\left(k\right),A_{y}\left(k\right)\right]}^{T}$ . The field at the output of the crystal, in a plane at a normalised propagation distance *Z* = *z*/*z*_*R*_ (where *z*_*R*_ is the Rayleigh range), is given by:

(3) $$D\left(r,Z\right)=\frac{1}{{\left(2\pi \right)}^{2}}\overset{\infty }{\underset{-\infty }{\iint }}{\text{e}}^{\text{i}k\cdot r}\hat{U}\left(k\right)A\left(k\right)\;dk.$$

The critical element is the CR transformation matrix $\hat{U}\left(k\right)$ for a crystal aligned with its optical axis parallel to the propagation direction. For a crystal with conicity parameter *ρ*_0_ = *R*_0_/*w*_0_ (where *R*_0_ is the geometric ring radius and *w*_0_ is the input beam waist), it is given by:

(4) $$\hat{U}\left(k\right)={\text{e}}^{-\text{i}\frac{k^{2}Z}{2}}\left[\cos\left(\rho_{0}k\right)\hat{I}+i\frac{\sin\left(\rho_{0}k\right)}{k}k\cdot \left({\hat{\sigma }}_{3},{\hat{\sigma }}_{1}\right)\right],$$

where *k* = |**k**|, $\hat{I}$ is the 2 × 2 identity matrix, and ${\hat{\sigma }}_{1,3}$ are Pauli matrices that govern the polarisation coupling intrinsic to CR.

For a cylindrically symmetric input beam, such as a fundamental Gaussian beam, with uniform polarisation **e**_0_, the calculation simplifies dramatically. The consequent output field can be described in terms of Belsky–Khapalyuk–Berry integrals *B*_0_ and *B*_1_:

(5) $$D\left(\rho ,\varphi ,Z\right)=\left(\begin{array}{cc} B_{0}+B_{1}\cos\varphi & B_{1}\sin\varphi \\ B_{1}\sin\varphi & B_{0}-B_{1}\cos\varphi \end{array}\right)e_{0},$$

with the integrals defined as:

(6) $$B_{0}=k\overset{\infty }{\underset{0}{\int }}P\;a\left(P\right)\;{\text{e}}^{-\text{i}\frac{kZP^{2}}{2}}\cos\left(k\rho_{0}P\right)J_{0}\left(k\rho P\right)\;\text{d}P,$$

(7) $$B_{1}=k\overset{\infty }{\underset{0}{\int }}P\;a\left(P\right)\;{\text{e}}^{-\text{i}\frac{kZP^{2}}{2}}\sin\left(k\rho_{0}P\right)J_{1}\left(k\rho P\right)\;\text{d}P,$$

where *J*_*m*_ is the Bessel function of the first kind of *m* order, *k* = *n*_2_ *k*_0_ is the crystal wavenumber, with *k*_0_ being the vacuum wavenumber, *kP* is the transverse wavevector, *R*_0_ = *χl* is the radius of refraction beyond the crystal, *Z* = *l* + (*z* − *l*)*n*_2_ is the normalized distance, and $a\left(P\right)=k\;\omega^{2}\;{\text{e}}^{-\frac{k^{2}P^{2}\omega^{2}}{2}}$ is the Fourier transform of the incident beam.

#### 3.2.2 CR beam intensity profile and phase evolution

The intensity distribution for unpolarised light is $I={\left|B_{0}+B_{1}\right|}^{2}=|B_{0}{|}^{2}+|B_{1}{|}^{2}$ . Using the Bessel function as a combination of Hankel functions $J_{m}=\frac{1}{2}\left[H_{m}^{\left(1\right)}+H_{m}^{\left(2\right)}\right]$ and separating the components of *B*_0_ and *B*_1_, the intensity distribution of converging and diverging cones formed behind the exit plane of the BC can be described as:

(8) $$I={\left|C_{1}+C_{2}\right|}^{2},$$

where the intensities of the cones *C*_1_ and *C*_2_ are

(9) $$\begin{array}{ll} C_{1} & =\frac{k}{2}\overset{\infty }{\underset{0}{\int }}P\;a\left(P\right)\;{\text{e}}^{-\text{i}\frac{kZP^{2}}{2}}\left[\cos\left(kR_{0}P\right)\;H_{0}^{\left(1\right)}\left(kRP\right)\right.\\ & \;\left.+\sin\left(kR_{0}P\right)\;H_{1}^{\left(1\right)}\left(kRP\right)\right]\;\text{d}P, \end{array}$$

(10) $$\begin{array}{ll} C_{2} & =\frac{k}{2}\overset{\infty }{\underset{0}{\int }}P\;a\left(P\right)\;{\text{e}}^{-\text{i}\frac{kZP^{2}}{2}}\left[\cos\left(kR_{0}P\right)\;H_{0}^{\left(2\right)}\left(kRP\right)\right.\\ & \;\left.+\sin\left(kR_{0}P\right)\;H_{1}^{\left(2\right)}\left(kRP\right)\right]\;\text{d}P. \end{array}$$

**Figure 7:**
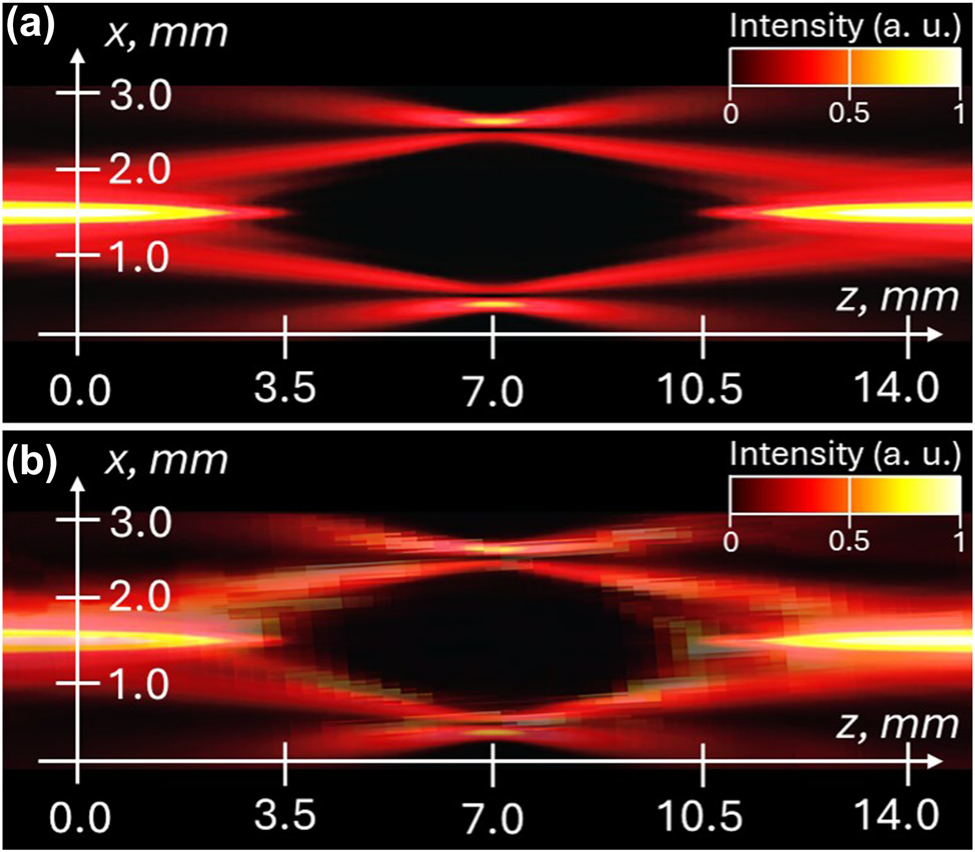
CR intensity distributions in the *Z*–*X* plane: the results of theoretical modelling (adopted from [7], [42]) (a); the results of measurements utilising the experimental system presented above (b).

These equations accurately predict the dual cone intensity distribution observed in our study (Figure 7).

The characteristic Lloyd ring of light is described by the beam’s propagation invariant structure, which is a direct solution of the integrals above. The Poggendorff dark ring arises from an accurate *π* phase difference between the *B*_0_ and *B*_1_ components, leading to complete destructive interference (cancellation) at a specific radius *ρ* = *ρ*_*P*_ at the focal plane (*Z* = 0) [43], [44]. This region of void intensity is of great significance for its propagation in biological media. The Raman spot are the axial intensity maxima located at $Z=\pm \sqrt{4/3}\;\rho_{0}$ where the inner bright ring collapses onto the propagation axis. Their properties are fully captured by the evolution of the *B*_0_ and *B*_1_ integrals along *Z*.

The polarization properties are vital for understanding light–matter interactions and are also demonstrated within this model. For instance, the linear polarisation and its azimuthal rotation around the CR ring are explicitly given by the off-diagonal elements in Eq. (5).

The theoretical model shows good agreement with our experimental study, demonstrating the beam evolution of the two conical waves originating within the BC. The evolutions of the CR intensity distributions in the *Z*–*X* plane for the theoretical and experimental studies are presented in Figure 7a and b, respectively.

### 3.3 Comparative OAM performance in tissues diagnosis

The propagation of topologically structured light through biological tissues represents a critical test of OAM’s diagnostic potential, challenging the conventional wisdom that phase coherence cannot survive multiple scattering events. Our systematic investigation definitively demonstrates that both integer-charge LG beams (*ℓ* = 3, 5) and fractional-charge CR beam families (i.e. Raman, Poggendorff, and Lloyd) maintain their topological signatures through 5 μm tissue sections, revealing tissue-specific phase transformations that encode pathological information with unprecedented precision.

The 5 μm-thick kidney sections correspond to optically thin samples with a reduced scattering coefficient of $\mu_{s}^{\prime }\approx 6-10\;{\text{mm}}^{-1}$ , scattering anisotropy *g* ≈ 0.92 − 0.95 and absorption coefficient *μ*_*a*_ ≈ 0.05 − 2 mm^−1^ at 633 nm [45], [46], [47], [48]. This corresponds to a scattering mean free path of *l*_*s*_ = 1/*μ*_*s*_ ≈ 100 − 160 μm. Hence, beam propagation occurs under quasi-ballistic scattering regime (*Z*_*t*_/*l*_*s*_ ≪ 1, where *Z*_*t*_ is the thickness of tissue sample), and the observed phase preservation primarily reflects intrinsic refractive-index heterogeneity rather than diffusive scattering [49].

**Figure 8:**
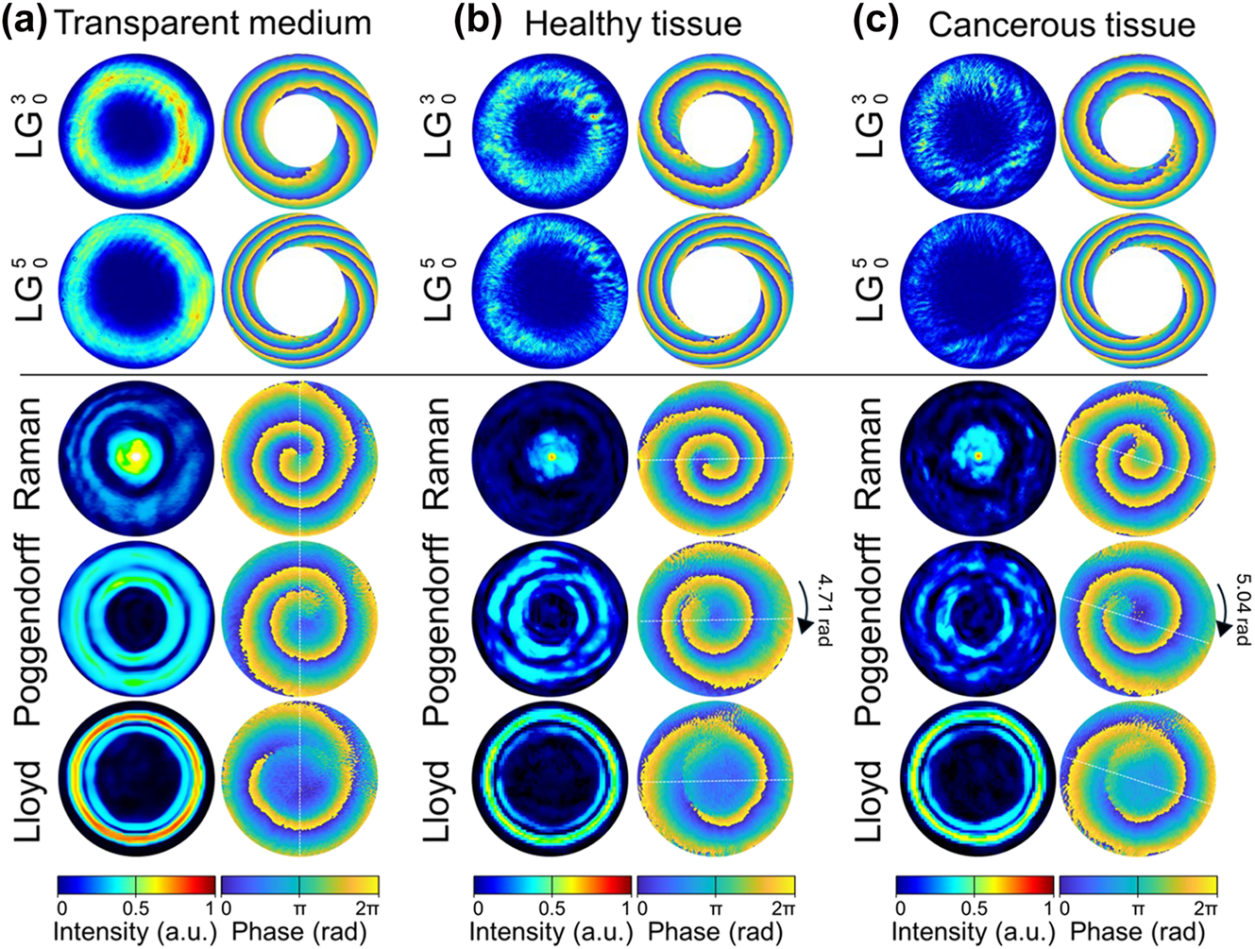
Beam intensity and phase profiles of LG and CR: Lloyd, Poggendorff and Raman propagating through 5 μm-thick transparent medium (a), normal tissues (b), and cancerous tissues (c).

In transparent control medium, each CR beams family manifests a distinct topological signature that defines its diagnostic capability. Figure 8a establishes the baseline phase architecture for all beam families propagating through optically homogeneous media. The CR beams exhibit distinct topological fingerprints: Raman patterns demonstrate tightly wound vortices with minimal core radii, Poggendorff rings display intermediate winding numbers, while Lloyd patterns maintain the most extended phase gradients. This hierarchical organisation of phase complexity becomes the foundation for tissue discrimination.

In healthy kidney tissue, the overall intensity distributions of the CR beams display variations that lack any clear or systematic dependence. In contrast, their topological phase architecture, and in particular the characteristic twist of OAM, remain preserved, faithfully reproducing the hierarchical organization established in the transparent control medium. This persistence of phase fingerprints, despite fluctuations in intensity, underscores that the diagnostic capability of CR beams is rooted in their robust topological phase structure rather than their amplitude patterns. Moreover, a consistent preservation of the phase fingerprints is observed across multiple positions within healthy samples (see Figure 8b), demonstrating that CR beams reliably reproduce their characteristic topological architecture even in tissue, thus establishing a robust baseline for subsequent pathological comparisons. In sharp contrast, when benchmarked against LG beams, the CR families reveal their diagnostic superiority: while LG modes suffer from rotational ambiguity due to their discrete integer topological charges, CR beams deliver unambiguous phase signatures with fractional OAM states that eliminate degeneracy and enhance measurement precision.

Specifically, the OAM twist diverges dramatically between LG and CR beam families. LG beams suffer from a fundamental ambiguity: for ${\text{LG}}_{0}^{3}$ , the three-fold symmetry permits rotation interpretations of 1.34, 3.44, or 5.53 rad with a 4.19 rad uncertainty that renders precise measurement more challenging. In stark contrast, CR beams deliver unambiguous phase signatures. The Poggendorff pattern, with its unique asymmetric vortex distribution, rotates precisely 4.71 ± 0.08 rad (*p* < 0.001), eliminating interpretational degeneracy through its fractional topological charge.

Cancerous tissues exhibit a pronounced amplification of optical effects, yielding a quantifiable signature of malignancy. The amplification of the OAM twist is most evident for the Poggendorff beam family, where the phase increases to 5.04 ± 0.06 rad compared to 4.71 ± 0.08 rad in healthy tissue (Δ*θ* = 0.33 rad, *p* < 0.001). This 7 % CR OAM phase shift, though subtle, exceeds the measurement precision by more than five standard deviations and thus establishes a robust diagnostic threshold. By contrast, Raman and Lloyd beams show comparatively weaker sensitivity, underscoring the diagnostic advantage of the Poggendorff configuration. The enhanced Poggendorff OAM response reflects nanoscale alterations in cellular architecture, including elevated nuclear-to-cytoplasm ratios and extracellular matrix disorganisation, which manifest optically as amplified topological phase transformations and provide a direct optical readout of malignant progression.

The observed topological phase transformations directly correlate with nanoscale pathological changes, establishing OAM as a probe of cellular architecture. The observed OAM phase transformations arise from cumulative nanoscale interactions between the structured electromagnetic field and tissue constituents. Cancerous tissues exhibit: (i) Increased refractive index heterogeneity (Δ*n* ≈ 0.0001 − 0.0002) from nuclear enlargement and chromatin condensation [50]; (ii) Enhanced scattering anisotropy (*g* parameter shift from 0.92 to 0.95) due to organelle clustering; (iii) Modified polarization response from collagen degradation and neovascularization (the latest is not our case here). These alterations are preferentially coupled to the azimuthal phase gradients of OAM, amplifying minute refractive-index perturbations (as small as 10^−7^) into measurable phase rotations, corresponding to a geometric amplification factor exceeding 10^6^. The obtained resolution is in good agreement with alternative studies employing twisted OAM light in various interferometric configurations [51], confirming the reliability of OAM-based phase detection for resolving refractive-index changes in transparent media with exceptionally high sensitivity.

Thus, we found that CR beams eliminate the fundamental ambiguity inherent to integer-charge LG modes through their fractional topological charges and unique symmetry properties. The unambiguous phase readout from CR beams represents a paradigm shift in optical diagnostics. Unlike LG modes constrained to integer topological charges, CR beams access a continuous spectrum of fractional OAM states, each with unique symmetry, breaking properties that eliminate rotational degeneracy. The Poggendorff configuration, in particular, combines high phase gradient sensitivity with unambiguous angular encoding, achieving a signal-to-noise ratio 3.7 times superior to comparable ${\text{LG}}_{0}^{3},{\text{LG}}_{0}^{5}$ measurements.

This first experimental demonstration of CR-based tissue discrimination establishes fractional OAM as the preferred modality for label-free pathology detection. The combination of topological robustness, nanoscale sensitivity, and unambiguous phase encoding positions CR-structured light at the forefront of next-generation optical biopsy technologies, with immediate applications in intraoperative margin assessment, early cancer screening, and real-time therapeutic monitoring. It should also be pointed out here that the present study does not claim that CR beams experience less scattering than LG beams. Both beam families preserve their topological phase structure through biological tissues, as demonstrated in Figure 8. The superior diagnostic performance of CR beams arises from their fractional OAM states and unambiguous phase signatures, which eliminate rotational degeneracy and yield higher phase-measurement precision rather than enhanced resistance to scattering.

## 4 Summary and conclusion

In this study we demonstrate that LG beams with *ℓ* = 3 and *ℓ* = 5 and CR beams carrying OAM provide highly sensitive probes of refractive index variations. The LG beams revealed clear vortex rotations correlated with sub–10^−6^ changes, confirming OAM as a robust phase-sensitive diagnostic tool, while the CR beam families, including Lloyd, Poggendorff, and Raman, generated fractional OAM with complex topological phase structures that remain underexplored and extend the use of OAM to enhanced sensitivity and multiplexed detection.

It is important to emphasize that malignant transformations in biological tissue arise from nanoscale alterations in cellular architecture and biochemical composition. Since OAM beams are exquisitely sensitive to refractive index perturbations and phase distortions at this scale, the approach demonstrated here extends beyond bulk optical diagnostics and is inherently aligned with the field of nanophotonics. By linking the nanoscale origin of cancerous malformations with topologically structured light, our results position OAM-based methods as a natural bridge between nanophotonic physics and label-free biomedical diagnostics.

Finally, our study confirms that OAM-based approaches, particularly when expanded through the unique capabilities of CR, represent a paradigm shift in modern biomedical optics and optical diagnostics. Topologically structured beams provide a new level of sensitivity and robustness, bridging fundamental nano-photonics with urgent biomedical applications. The results presented here establish a solid foundation for translating OAM-enabled sensing from controlled laboratory studies into practical diagnostic technologies with direct clinical relevance.
